# Hypertensive Disorders of Pregnancy in Patients With Cardiac Disease: Risk and Impact on Delivery Timing

**DOI:** 10.1016/j.jacadv.2025.102130

**Published:** 2025-09-15

**Authors:** Maura Jones Pullins, Johanna Quist-Nelson, Matthew Fuller, Elizabeth Volz, Sarah Snow, Ashraf S. Habib, Jerome Federspiel, Kim Boggess, Marie-Louise Meng

**Affiliations:** a Division of Maternal-Fetal Medicine, Department of Obstetrics and Gynecology, University of North Carolina at Chapel Hill, Chapel Hill, North Carolina, USA; b Department of Biostatistics and Bioinformatics, Duke University, Durham, North Carolina, USA; c Division of Cardiology, Department of Medicine, University of North Carolina at Chapel Hill, Chapel Hill, North Carolina, USA; d Division of Cardiology, Department of Medicine, Duke University School of Medicine, Durham, North Carolina, USA; e Department of Anesthesiology, Duke University School of Medicine, Durham, North Carolina, USA; f Division of Maternal-Fetal Medicine, Department of Obstetrics and Gynecology, Duke University School of Medicine, Durham, North Carolina, USA; g Division of Hematology, Department of Medicine, Duke University School of Medicine, Durham, North Carolina, USA; h Department of Population Health Sciences, Duke University School of Medicine, Durham, North Carolina, USA

**Keywords:** cardiovascular disease, hypertensive disorders of pregnancy, pre-eclampsia

## Abstract

**Background:**

Pregnant patients with cardiovascular disease (CVD) face increased risk of hypertensive disorders of pregnancy (HDP) and preterm delivery, yet data are limited regarding the degree of risk and impact of HDP on gestational age at delivery.

**Objectives:**

The objective of the study was to examine the HDP risk and impact on gestational age at delivery in patients with CVD.

**Methods:**

This retrospective cohort study used the Premier Healthcare Database and included patients >18 years who delivered between October 1, 2015, and December 31, 2020. Patients with CVD were categorized into the following 6 subtypes: congenital, ischemic, aortic pathology, pulmonary hypertension (HTN), cardiomyopathy, and valvular disease. Primary outcome was odds of HDP (gestational HTN or pre-eclampsia); Secondary outcome was gestational age at delivery. Multivariable mixed effects regression models were used to estimate adjusted outcomes, adjusting for CVD subtype, >1 CVD subtype present, demographics, hospital characteristics, and comorbidities.

**Results:**

Among 4,606,247 obstetric patients, 20,021 had CVD. HDP risk varied by CVD subtype, lowest in those with congenital heart disease (adjusted OR [aOR]: 0.9; 95% CI: 0.8-1.0) and highest in those with pulmonary HTN (aOR: 1.5; 95% CI: 1.3-1.8) and cardiomyopathy (aOR: 1.5; 95% CI: 1.4-1.6). Patients with CVD delivered earlier than those without CVD, even in the absence of HDP (36.4-38.0 weeks vs 38.4 weeks). Among those with HDP, patients with severe pre-eclampsia with CVD, delivered earlier than those without CVD (33.1-34.6 weeks vs 35.5 weeks).

**Conclusions:**

Odds of HDP and delivering at an earlier gestational age differs across CVD subtypes, notably higher in those with pulmonary HTN and cardiomyopathy, emphasizing the need for individualized counseling.

## Background

Pre-eclampsia complicates 2% to 8% of pregnancies and is a leading cause of maternal morbidity and medically indicated preterm birth in the United States.^1^ Similarly, cardiovascular disease (CVD) is a leading cause of maternal morbidity and mortality, with increasing prevalence, particularly of acquired cardiac disease, in pregnant patients in the United States.^2^^,^^3^

Among patients with asymptomatic CVD, studies indicate that early-term delivery (delivery between 37-38 weeks of gestation) does not improve maternal cardiovascular or obstetric outcomes but does increase adverse neonatal outcomes when compared with delivery at 39 weeks gestation.^4^, ^5^, ^6^ In line with this, the American College of Obstetricians and Gynecologists (ACOG) recommends delivery at 39 weeks in those with asymptomatic cardiac disease but does not make additional recommendations regarding delivery timing in this patient population, deferring to the managing pregnancy heart team. Conversely, in patients with hypertensive disorders of pregnancy (HDP), recommendations regarding delivery timing are well-delineated by ACOG and oftentimes require preterm (34 0/7 weeks) or early-term birth (37 0/7 weeks).^7^

Patients with cardiac disease are at an increased risk of both HDP as well as preterm delivery.^8^ Pre-eclampsia can precipitate cardiovascular decompensation in previously stable or asymptomatic patients with CVD, increasing the risk of heart failure, cesarean delivery, preterm birth, small for gestational age neonates, and even maternal and perinatal death.^8^ However, the impact of HDP on delivery timing and thus gestational age at delivery, has not been well characterized in this population, nor has gestational age at delivery been examined by the specific CVD subtype. Clarifying these overlapping risk profiles is critical for guiding management and counseling patients about potential complications and delivery decisions.

Given the limited data on delivery timing in patients with CVD who develop HDP, our study aimed to describe the odds of HDP according to the CVD subtype. We also sought to characterize how developing HDP affects delivery timing in patients with different types of CVD in the United States—specifically detailing the gestational week of delivery, as this greatly impacts neonatal morbidity.^9^^,^^10^

## Methods

### Study design and population

This retrospective cohort study used the Premier Healthcare Database (Premier Inc). Details of the data set and readmission data have been previously described.^11^ The cohort for this study consisted of pregnant patients 18 years of age or older delivering between October 1, 2015 and December 31, 2020, who had both an International Classification of Diseases-10th edition (ICD-10) diagnosis code for delivery after 25 weeks gestation (Z3A.25-Z3A.42) and for cardiac disease (Supplemental Table 1). This study was deemed exempt from review by the Duke University Health System Institutional Review Board and follows the Strengthening the Reporting of Observational Studies in Epidemiology guidelines.^12^

### Exposures, outcomes, and covariates

The exposure variable of interest was cardiac disease, which was divided into the following 6 subtypes: aortic, ischemic, congenital, pulmonary hypertension (HTN), cardiomyopathy, and valvular disease (Supplemental Table 1). Patients with codes for multiple subtypes of CVD were included in the analyses for each of these CVD categories.

The outcome of interest was a diagnosis of an HDP: gestational HTN, pre-eclampsia or pre-eclampsia with severe features (SF) at delivery hospitalization. These categories were considered mutually exclusive and were ascertained by diagnosis codes for gestational HTN and pre-eclampsia disorders (Supplemental Table 1). If a patient had multiple HDP codes, they were placed in the more severe disease category (ie, least severe: gestational HTN, followed by pre-eclampsia without SF, followed by pre-eclampsia with SF).

The primary outcome was prevalence and odds of HDP in patients with CVD, stratified by the CVD subtype. The secondary outcome was the gestational age at delivery by the CVD subtype and type of HDP.

The covariates in the models were selected a priori based on known maternal morbidity risk factors, prior literature, and subject-matter expertise: age, payor category (managed care organization, Medicaid, Medicare, and other), race/ethnicity, urban vs rural, hospital teaching status, hospital size, hospital region, and the presence of more than one category of cardiac disease. Comorbid conditions were defined using the Leonard Expanded Obstetric Comorbidity Index, with modifications (Supplemental Table 2), and were included as covariates in all multivariable models to adjust for maternal health status at delivery admission. In contrast to standard use of the index, we did not include pre-eclampsia or pre-eclampsia with SF as covariates, as they were exposure variables in our analysis.^13^ Importantly, chronic HTN was treated as a comorbidity, not as a type of HDP.

Diagnosis codes were accompanied by a “present on admission” (POA) flag, indicating whether the condition was present at the time of hospital admission. POA flag values include: Y (yes, present on admission), N (no, developed during hospitalization), U (documentation insufficient), W (clinically undetermined), and blank (not subject to POA reporting). This flag was used to distinguish pre-existing conditions from hospital-acquired diagnoses. When POA status was uncertain (U, W, or blank), diagnoses were treated as present on admission.

Some heart failure ICD-10 codes represent chronic disease with an acute component (Supplemental Table 1) and could serve as both inclusion criteria and outcomes. To avoid misclassification, these codes were excluded as inclusion criteria in the primary analysis and treated as outcomes only when not present on admission.

In a planned sensitivity analysis, we evaluated the impact of including 1) diagnoses with uncertain POA status as not present on admission, and 2) acute-on-chronic heart failure codes as inclusion criteria regardless of the POA status.

### Statistical analysis

Demographic and clinical characteristics of the cohort are reported, stratified by the subtype of CVD. Descriptive statistics were used to examine the study population with categorical variables reported as counts and frequencies, and continuous data reported as median (IQR). A multivariable logistic regression model was used to compare the association between the CVD subtype and the development of HDP. This regression model included fixed effects for patient and hospital characteristics and a random intercept for hospitals. The association between the CVD subtype and the development of HDP are reported as ORS with 95% CIs. Associations between CVD categories, HDP, and gestational age at delivery were assessed using a multivariable mixed linear regression model. This model included fixed effects for patient and hospital characteristics and a random intercept for each hospital to account for the clustering of patients within centers. The adjusted mean gestational age at delivery was estimated using this model and is reported with an associated 95% CI.

Statistical analyses were performed in the SAS System (version 9.4, SAS Institute). A 2-sided *P* value <0.05 was prespecified as statistically significant.

## Results

Of 4,606,247 deliveries, 20,021 (0.43%) deliveries had a diagnosis of CVD present on admission. Baseline demographic characteristics and comorbidities are described in Table 1. The median age among patients with CVD was 31 years (Q1-Q3: 25-34) and 29 years (Q1-Q3: 25-33) in those without CVD. Most patients with CVD delivered in urban, large, or teaching hospitals. Patients with CVD were more likely to have chronic HTN; ranging from 6.7% in those with congenital heart disease to 30.5% in those with cardiomyopathy, compared to 3.4% in those without CVD.

**Table 1:** Baseline Demographic Characteristics of Cohort

Cardiac Disease Classification	Aortic Pathology (n = 1,080)	Ischemic (n = 1,696)	Congenital (n = 5,101)	Pulmonary HTN (n = 648)	Cardiomyopathy (n = 3,529)	Valvular (n = 8,771)	No Cardiac Pathology (n = 4,607,497)
Demographic characteristics							
Age, y, median [Q1, Q3]	29 [25, 34]	32 [28, 36]	28 [24, 33]	31 [27, 35]	31 [27, 36]	31 [26, 35]	29 [25, 33]
Primary payor							
Managed care	453 (41.9%)	458 (27.0%)	1943 (38.1%)	163 (25.2%)	961 (27.2%)	3,820 (43.6%)	1,817,368 (39.4%)
Medicaid	372 (34.4%)	942 (55.5%)	2,133 (41.8%)	371 (57.3%)	1947 (55.2%)	3,090 (35.2%)	1,954,644 (42.4%)
Medicare	27 (2.5%)	65 (3.8%)	89 (1.7%)	31 (4.8%)	150 (4.3%)	135 (1.5%)	28,027 (0.6%)
Other	228 (21.1%)	231 (13.6%)	936 (18.3%)	83 (12.8%)	471 (13.3%)	1726 (19.7%)	807,458 (17.5%)
Race							
Asian	11 (1.0%)	33 (1.9%)	183 (3.6%)	21 (3.2%)	114 (3.2%)	281 (3.2%)	214,627 (4.7%)
Black	85 (7.9%)	378 (22.3%)	590 (11.6%)	210 (32.4%)	1,111 (31.5%)	1,034 (11.8%)	667,419 (14.5%)
Other	75 (6.9%)	174 (10.3%)	536 (10.5%)	96 (14.8%)	390 (11.1%)	871 (9.9%)	607,762 (13.2%)
Unknown	21 (1.9%)	45 (2.7%)	171 (3.4%)	25 (3.9%)	112 (3.2%)	227 (2.6%)	194,621 (4.2%)
White	888 (82.2%)	1,066 (62.9%)	3,621 (71.0%)	296 (45.7%)	1802 (51.1%)	6,358 (72.5%)	2,923,068 (63.4%)
Ethnicity							
Hispanic	86 (8.0%)	176 (10.4%)	627 (12.3%)	87 (13.4%)	390 (11.1%)	815 (9.3%)	726,482 (15.8%)
Non-Hispanic	804 (74.4%)	1,193 (70.3%)	3,460 (67.8%)	417 (64.4%)	2,445 (69.3%)	6,171 (70.4%)	3,029,385 (65.7%)
Unknown	190 (17.6%)	327 (19.3%)	1,014 (19.9%)	144 (22.2%)	694 (19.7%)	1785 (20.4%)	851,630 (18.5%)
Hospital characteristics							
Urban hospital	989 (91.6%)	1,546 (91.2%)	4,632 (90.8%)	596 (92.0%)	3,224 (91.4%)	7,976 (90.9%)	4,059,322 (88.1%)
Teaching hospital	690 (63.9%)	1,036 (61.1%)	3,214 (63.0%)	479 (73.9%)	2,315 (65.6%)	5,206 (59.4%)	2,189,591 (47.5%)
Bed size							
000-099	39 (3.6%)	64 (3.8%)	242 (4.7%)	10 (1.5%)	86 (2.4%)	350 (4.0%)	252,865 (5.5%)
100-199	92 (8.5%)	140 (8.3%)	448 (8.8%)	32 (4.9%)	213 (6.0%)	899 (10.2%)	666,751 (14.5%)
200-299	103 (9.5%)	193 (11.4%)	562 (11.0%)	57 (8.8%)	378 (10.7%)	1,040 (11.9%)	730,152 (15.8%)
300-399	126 (11.7%)	248 (14.6%)	659 (12.9%)	88 (13.6%)	528 (15.0%)	1,287 (14.7%)	807,266 (17.5%)
400-499	198 (18.3%)	225 (13.3%)	751 (14.7%)	87 (13.4%)	484 (13.7%)	1,198 (13.7%)	596,451 (12.9%)
500+	522 (48.3%)	826 (48.7%)	2,439 (47.8%)	374 (57.7%)	1840 (52.1%)	3,997 (45.6%)	1,554,012 (33.7%)
Region of country							
Midwest	283 (26.2%)	401 (23.6%)	1,153 (22.6%)	148 (22.8%)	827 (23.4%)	1808 (20.6%)	1,001,867 (21.7%)
Northeast	158 (14.6%)	245 (14.4%)	867 (17.0%)	110 (17.0%)	461 (13.1%)	1741 (19.8%)	670,286 (14.5%)
South	472 (43.7%)	794 (46.8%)	2,204 (43.2%)	294 (45.4%)	1,636 (46.4%)	3,972 (45.3%)	2,105,715 (45.7%)
West	167 (15.5%)	256 (15.1%)	877 (17.2%)	96 (14.8%)	605 (17.1%)	1,250 (14.3%)	829,629 (18.0%)
Mode of delivery							
Vaginal delivery	493 (45.6%)	743 (43.8%)	2,798 (54.9%)	195 (30.1%)	1,291 (36.6%)	4,628 (52.8%)	2,937,916 (63.8%)
Operative vaginal delivery	85 (7.9%)	77 (4.5%)	240 (4.7%)	27 (4.2%)	140 (4.0%)	421 (4.8%)	173,615 (3.8%)
Intrapartum CD	116 (10.7%)	271 (16.0%)	713 (14.0%)	136 (21.0%)	674 (19.1%)	1,149 (13.1%)	526,189 (11.4%)
Nonintrapartum CD	386 (35.7%)	605 (35.7%)	1,350 (26.5%)	290 (44.8%)	1,424 (40.4%)	2,573 (29.3%)	969,777 (21.0%)
Chronic HTN	139 (12.9%)	436 (25.7%)	343 (6.7%)	163 (25.2%)	1,077 (30.5%)	853 (9.7%)	155,493 (3.4%)
HDP	183 (16.9%)	411 (24.2%)	749 (14.7%)	251 (38.7%)	1,236 (35.0%)	1,527 (17.4%)	547,064 (11.9%)
Gestational HTN	84 (7.8%)	124 (7.3%)	35 (7.2%)	49 (7.6%)	263 (7.5%)	582 (6.6%)	290,551 (6.3%)
Pre-eclampsia without SF	62 (5.6%)	189 (10.2%)	238 (4.6%)	118 (15.8%)	702 (15.2%)	531 (6.0%)	163,485 (3.5%)
Pre-eclampsia with SF	42 (3.8%)	164 (8.9%)	191 (3.7%)	130 (17.4%)	851 (18.4%)	529 (5.9%)	121,777 (2.6%)
HELLP syndrome^a^	4 (0.4%)	27 (1.5%)	21 (0.4%)	14 (1.9%)	119 (2.6%)	77 (0.9%)	12,779 (0.3%)
Preterm labor	61 (5.6%)	137 (8.1%)	309 (6.1%)	85 (13.1%)	425 (12.0%)	511 (5.8%)	197,866 (4.3%)
Preterm PROM	38 (3.5%)	75 (4.4%)	223 (4.4%)	23 (3.5%)	167 (4.7%)	362 (4.1%)	135,548 (2.9%)

CD = cesarean delivery; HDP = hypertensive disorders of pregnancy; HELLP syndrome = hemolysis, elevated liver enzymes, low platelets syndrome; HTN = hypertension; PROM = prelabor rupture of membranes; SF = severe feature.

a HELLP syndrome included in pre-eclampsia with SF.

### Prevalence and odds of hypertensive disorders of pregnancy

**Figure 1:**
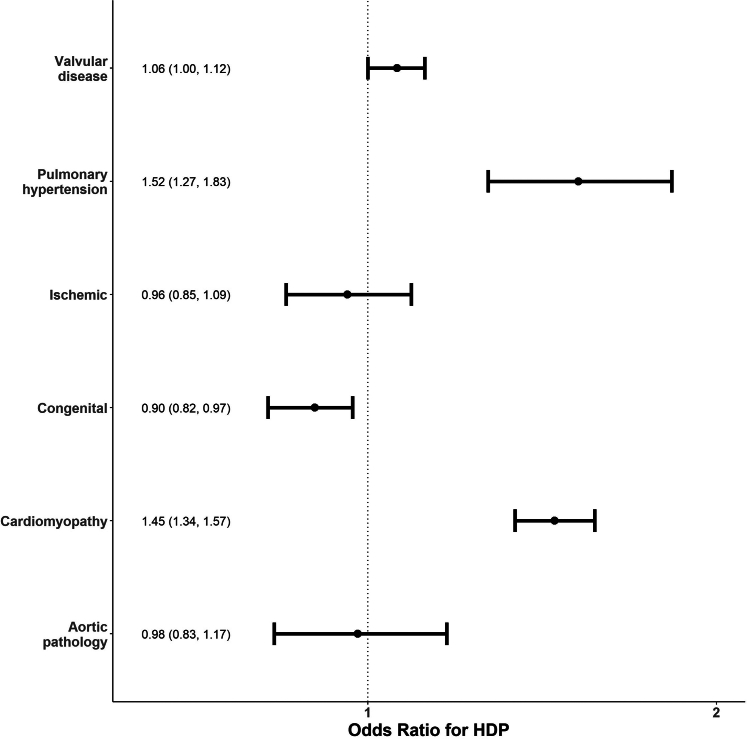
**Adjusted Odds Ratio of HDP by CVD Subtype** Adjusted odds ratio of hypertensive disorder of pregnancy, by cardiac disease subtype. Reference group for each subtype are those without that CVD subtype diagnosis code. CVD = cardiovascular disease; HDP = hypertensive disorders of pregnancy.

The prevalence of HDP in patients without CVD was 11.9%. The rate of HDP among patients with CVD varied by the CVD subtype, with the lowest rate in patients with congenital CVD at 14.7% and the highest in those with pulmonary HTN at 38.7%, cardiomyopathy at 35.0%, and ischemic cardiac disease at 24.2% (Table 1). After adjustment for age, payor, race, ethnicity, hospital type, hospital location, hospital size, and comorbidities (including chronic HTN), the odds of HDP were slightly lower in those with congenital CVD (OR: 0.90; 95% CI: 0.82-0.97; *P* = 0.009) and significantly higher in those with pulmonary HTN (OR: 1.5; 95% CI: 1.3-1.8; *P* < 0.001) and cardiomyopathy (OR: 1.5; 95% CI: 1.4-1.6; *P* < 0.001) relative to patients without CVD and without the specific CVD subtype being analyzed (Figure 1).

### Gestational age at delivery

**Figure 2:**
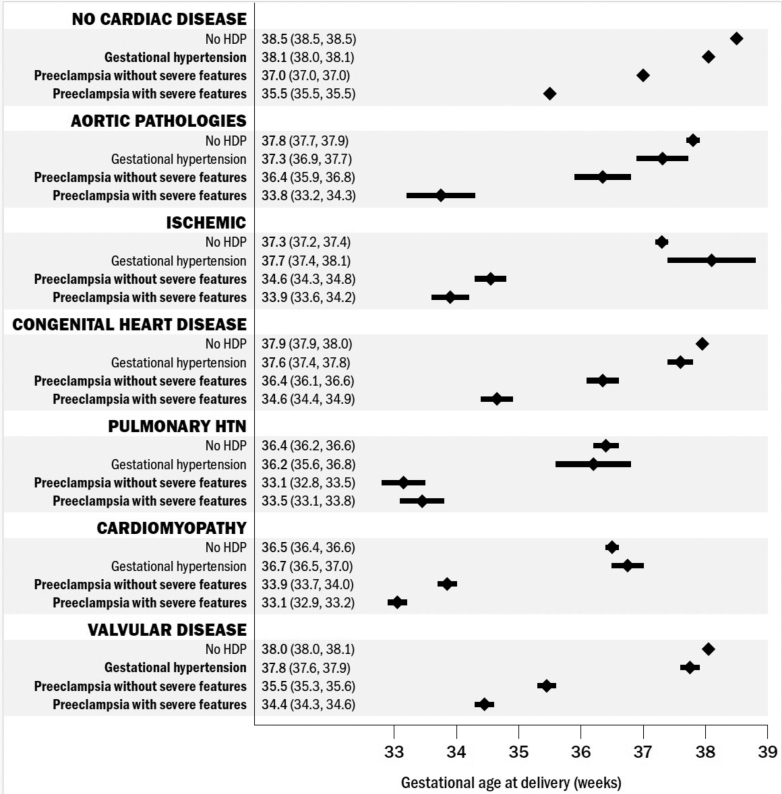
**Gestational Age at Delivery by HDP in Those With CVD** Adjusted mean gestational age at delivery among patients with CVD, stratified by CVD and HDP subtype. HTN = hypertension; other abbreviations as in Figure 1.

Gestational age at delivery in this cohort is described in Figure 2 and is presented as adjusted mean gestational ages reported with an associated 95% CI. On average, patients with CVD delivered at earlier gestational ages across the 6 subtypes (adjusted means ranging from 36.4-38.0 weeks) than those without CVD (38.4 weeks), even in the absence of concomitant HDP. Patients with HDP and CVD, across all subtypes, delivered between 33 and 37 weeks (adjusted means ranging from 33.1-37.8 weeks) whereas patients with HDP without CVD delivered at 37 weeks (37.2 weeks). When examining by HDP type, patients with gestational HTN and CVD, across all subtypes, delivered at 36 to 37 weeks (adjusted means ranging from 36.2-37.8 weeks) vs at 38 weeks (38.1 weeks) in patients with gestational HTN without CVD. Patients with pre-eclampsia without SF and CVD, across all subtypes, delivered at 33 to 36 weeks (adjusted means ranging from 33.1 to 36.4 weeks) vs 37 weeks (36.9 weeks) in patients with pre-eclampsia without SF, without CVD. Patients with pre-eclampsia with SF and CVD, across all subtypes, delivered at 33 to 34 weeks (adjusted means ranging from 33.1 – 34.6 weeks) vs 35 weeks (35.5 weeks) in patients with pre-eclampsia with SF, without CVD.

Across all CVD subtypes, those diagnosed with pre-eclampsia with SF delivered 3 to 4 weeks earlier than those within their same CVD subtype who did not develop HDP (Table 2). Patients with pulmonary HTN or cardiomyopathy delivered earliest regardless of HDP diagnosis. Patients with pulmonary HTN without HDP delivered at 36 weeks (36.4 weeks) however if diagnosed with pre-eclampsia with SF, the adjusted mean gestational age at delivery was 33 weeks (33.4 weeks). Similarly, those with cardiomyopathy delivered at 36 weeks (36.5 weeks) if they did not develop HDP vs 33 weeks (33.1 weeks) if they developed pre-eclampsia with SF (Figure 2).

**Table 2:** Gestational Age at Delivery by Hypertensive Disorder of Pregnancy in Those With Cardiac Disease, by Subtype

Cardiac Disease Subtype	Adjusted Mean Delivery Time (95% CI), weeks	Difference (No HDP – HDP) (95% CI), weeks	*P* Value
No cardiac disease			
No HDP	38.48 (38.46-38.49)	(reference)	
Gestational HTN	38.05 (38.03-38.07)	0.43 (0.42-0.44)	<0.001
Pre-eclampsia	36.98 (36.96-37.01)	1.49 (1.48-1.50)	<0.001
Pre-eclampsia with SF	35.51 (35.49-35.54)	2.96 (2.95-2.97)	<0.001
Aortic pathologies			
No HDP	37.79 (37.67-37.91)	(reference)	
Gestational HTN	37.33 (36.94-37.73)	0.45 (0.05-0.86)	0.030
Pre-eclampsia	36.39 (35.94-36.84)	1.40 (0.94-1.86)	<0.001
Pre-eclampsia with SF	33.79 (33.23-34.34)	4.00 (3.43-4.57)	<0.001
Ischemic			
No HDP	37.34 (37.24-37.44)	(reference)	
Gestational HTN	37.74 (37.40-38.08)	−0.40 (−0.75 to −0.04)	0.028
Pre-eclampsia	34.56 (34.29-34.82)	2.78 (2.50-3.07)	<0.001
Pre-eclampsia with SF	33.87 (33.56-34.17)	3.47 (3.15-3.79)	<0.001
Congenital disorders			
No HDP	37.92 (37.86-37.97)	(reference)	
Gestational HTN	37.61 (37.42-37.81)	0.30 (0.10-0.50)	0.003
Pre-eclampsia	36.36 (36.13-36.59)	1.56 (1.33-1.79)	<0.001
Pre-eclampsia with SF	34.63 (34.37-34.88)	3.29 (3.03-3.55)	<0.001
Pulmonary HTN			
No HDP	36.41 (36.23-36.58)	(reference)	
Gestational HTN	36.18 (35.60-36.76)	0.23 (−0.38 to 0.83)	0.462
Pre-eclampsia	33.14 (32.80-33.47)	3.27 (2.89-3.65)	<0.001
Pre-eclampsia with SF	33.48 (33.13-33.83)	2.93 (2.54-3.31)	<0.001
Cardiomyopathy			
No HDP	36.51 (36.44-36.59)	(reference)	
Gestational HTN	36.74 (36.48-37.00)	−0.23 (−0.50 to 0.04)	0.094
Pre-eclampsia	33.87 (33.72-34.02)	2.64 (2.48-2.81)	<0.001
Pre-eclampsia with SF	33.06 (32.90-33.21)	3.46 (3.29-3.63)	<0.001
Valvular disease			
No HDP	38.02 (37.98-38.07)	(reference)	
Gestational HTN	37.77 (37.61-37.93)	0.26 (0.10-0.42)	0.002
Pre-eclampsia	35.46 (35.31-35.62)	2.56 (2.40-2.72)	<0.001
Pre-eclampsia with SF	34.41 (34.25-34.56)	3.62 (3.46-3.78)	<0.001

Abbreviations as in Table 1.

## Discussion

This large retrospective study of obstetric patients found that the odds of HDP varies by the CVD subtype (Central Illustration). Significantly higher odds of HDP were observed in individuals with pulmonary HTN and cardiomyopathy, whereas congenital heart disease was associated with lower odds of HDP. No significant associations were found for valvular disease, ischemic heart disease, or aortic pathology. Patients with CVD delivered at earlier gestational ages than those without CVD, irrespective of the presence of HDP (Central Illustration). When HDP were present, patients with CVD delivered 2 to 3 weeks earlier compared to those without CVD and HDP. Among these, patients with pre-eclampsia with SF experienced the earliest deliveries. Notably, across all CVD subtypes, those diagnosed with pre-eclampsia with SF delivered 3 to 4 weeks earlier than those within their same CVD subtype who did not develop HDP.

**Central Illustration:**
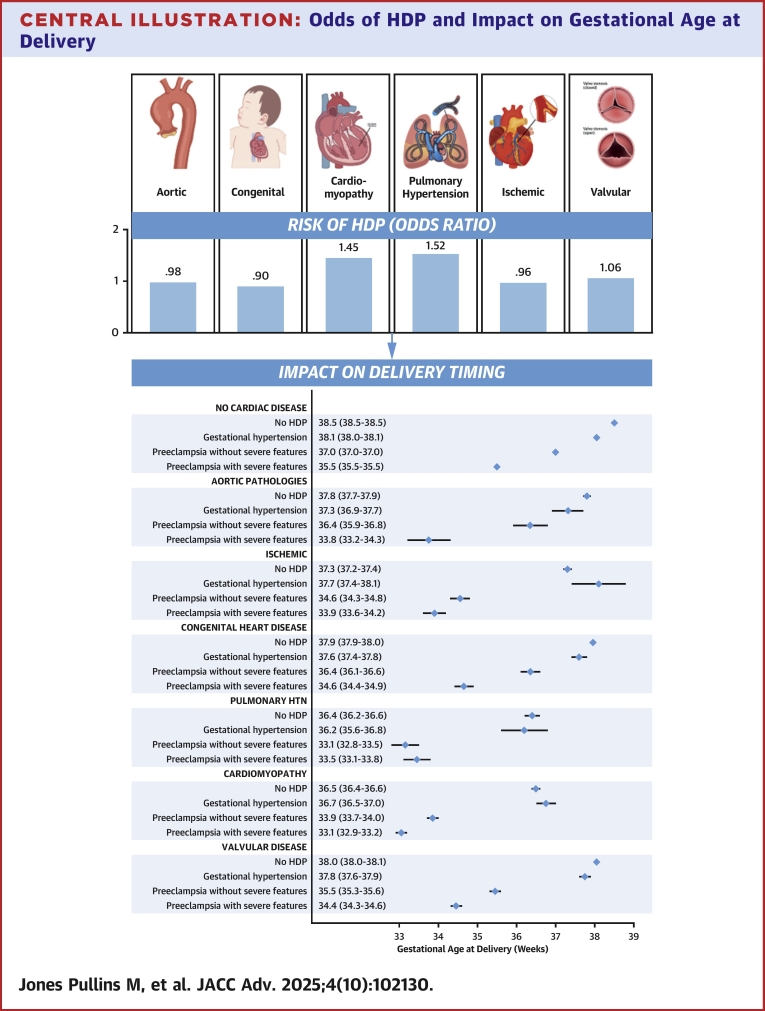
**Odds of HDP and Impact on Gestational Age at Delivery** Patients with CVD delivered at earlier gestational ages than those without CVD, even in the absence of HDP. When HDP was present, patients with CVD delivered 2 to 3 weeks earlier than those without CVD and HDP. Among all groups, patients with pre-eclampsia with severe features (SF) experienced the earliest deliveries. Across all CVD subtypes, those with pre-eclampsia with SF delivered 3 to 4 weeks earlier than peers within the same CVD subtype who did not develop HDP. CVD = cardiovascular disease; HDP = hypertensive disorders of pregnancy; HTN = hypertension.

The prevalence of CVD is rising among patients of childbearing age and remains a leading cause of maternal morbidity and mortality. Early, individualized counseling regarding maternal and fetal risks of pregnancy when complicated by CVD is essential. Low-dose aspirin should be recommended for pre-eclampsia prevention in patients at high risk of pre-eclampsia development regardless of the CVD subtype.^14^ Our results indicate that pregnant patients with CVD are more likely to deliver at an earlier gestational age, particularly if diagnosed with HDP. Although ACOG recommends patients with asymptomatic CVD deliver at 39 weeks, these data suggest that patients with CVD are frequently delivered early term, even in the absence of concomitant HDP.^6^ This trend may reflect the presence of maternal symptoms or fetal complications that necessitate earlier delivery in this high-risk population.

Furthermore, ACOG provides identical delivery timing recommendations for patients with pre-eclampsia without SF and patients with gestational HTN, both at 37 weeks; yet in our study, patients with pre-eclampsia without SF delivered, on average, earlier than those with gestational HTN, across all CVD subtypes.^1^ These findings suggest that within the U.S. clinicians may be unlikely to expectantly manage patients with pre-eclampsia without SF in the context of CVD until recommended delivery timing, or that patients with CVD who have pre-eclampsia without SF develop indications for earlier delivery.

Our findings support re-evaluating ACOG guidelines to include CVD—particularly cardiomyopathy and pulmonary HTN—as indications for prophylactic low-dose aspirin. Currently, aspirin is recommended for patients with at least one high-risk factor (eg, chronic HTN, diabetes) or 2 moderate-risk factors (eg, nulliparity, body mass index >30 kg/m^2^), but CVD is not among them.^14^ Low-dose aspirin is an evidence-based intervention; the ASPRE (Aspirin versus Placebo in Pregnancies at High Risk for Preterm Pre-eclampsia) trial demonstrated a 62% reduction in preterm pre-eclampsia with 150 mg initiated between 12 and 16 weeks in high-risk patients.^15^ Although support is growing for universal aspirin use due to the limited sensitivity of risk-factor–based screening and emerging evidence favoring doses of 150 to 162 mg, we propose that cardiomyopathy and pulmonary HTN be added to the list of risk factors until universal prophylaxis is more widely implemented.^16^, ^17^, ^18^, ^19^, ^20^

### Comparison to other studies

The primarily European registry-based study Hypertensive Disorders of pregnant women with heart disease: the ESC EORP Registry of Pregnancy and Cardiac Disease (ROPAC), included 5,700 pregnancies among individuals with CVD and reported a prevalence of 10.3% of HDP. A key difference in the ROPAC study is that they include chronic HTN in their definition of HDP, whereas our analysis treated chronic HTN as a separate comorbidity. Thus, when excluding chronic HTN to enable more direct comparison, the prevalence of HDP in the ROPAC study was 4.4%. They similarly observed the highest prevalence in those with pulmonary HTN (11.1%), cardiomyopathy (8.4%), and ischemic CVD (6.3%).^8^ In contrast, our U.S.-based study, observed an overall higher prevalence of HDP at 11.2%, with notably higher rates in patients with pulmonary HTN (38.7%), cardiomyopathy (35.0%), and ischemic CVD (24.2%).

Our findings are consistent with other U.S.-based studies. An analysis using the National Inpatient Sample reported an increased prevalence of pre-eclampsia among women with CVD. However, akin to the findings in our study, the rates of HDP in the National Inpatient Sample were notably higher, particularly among those with cardiomyopathy (25.6%) and pulmonary HTN (22.3%).^3^^,^^8^ Similarly, a New York-based study using ICD codes found comparable rates of HDP to the rates in our cohort (cardiomyopathy [25.3%] and pulmonary HTN [23.3%]).^21^ These data highlight the heightened prevalence of HDP in U.S. cohorts. Differences in prevalence compared to the ROPAC study may reflect broader inclusion criteria and reliance on administrative coding in U.S. datasets, vs ROPAC’s more focused recruitment of severe cases in a primarily European registry.^3^^,^^8^^,^^21^ Regional variations in population characteristics, health care systems, and clinical practices may also contribute to these discrepancies.

When considering the odds of HDP in patients with CVD, the ROPAC study similarly found that pulmonary HTN was associated with significantly increased odds of pre-eclampsia (OR: 1.7; 95% CI: 1.1-2.7). In our cohort patients with congenital heart disease had decreased odds of pre-eclampsia. This is consistent with prior literature which reported pre-eclampsia rates in this population comparable to the general obstetric population.^8^^,^^21^^,^^22^ Interestingly, although we observed no significant association between valvular heart disease and HDP, a Swedish cohort study reported an increased risk of pre-eclampsia in this group but found no increased risk among patients with heart failure.^23^ These findings underscore our central conclusion: CVD is a heterogeneous condition, with different subtypes demonstrating distinct pathophysiologic mechanisms, clinical courses, and relationships with HDP.

CVD is a well-established risk factor for preterm birth.^21^^,^^24^^,^^25^ However, the extent to which HDP contribute to this risk in patients with CVD has not been well studied. The ROPAC study reported significantly higher rates of preterm delivery among CVD patients with HDP (gestational HTN or pre-eclampsia)—34.1% compared to 16.9% in those without HDP. However, their analysis distinguished only between early-onset (<34 weeks) and late-onset (≥34 weeks) pre-eclampsia, without further detail on gestational age at delivery.^8^ Given that neonatal outcomes occur along a continuum—with each additional week of gestation conferring clinical benefit—our study offers a novel contribution by evaluating delivery timing by exact week of gestation.^26^^,^^27^ This more granular approach provides greater clinical insight and enables more precise, individualized counseling.

### Strengths

Our study has several strengths. The sample size, which was 4 times larger than that of the ROPAC study, provided sufficient power to detect differences in the odds of HDP and gestational age at delivery, and the specific evaluation of associations by the 6 most common CVD subtypes.^8^ Although we noted an increased prevalence of HDP among specific CVD subtypes, the overall prevalence of pre-eclampsia in our study was 5.8%, compared to the general population estimate of 2% to 8% for pre-eclampsia, indicating the Premier Healthcare Database is representative of the U.S. population.

### Study Limitations

As with many large administrative databases, there is potential for misclassification of diagnoses, particularly for complex conditions, with studies indicating misclassification results in underestimation of prevalence, particularly for pre-eclampsia.^9^^,^^10^ ICD codes may not capture all clinical nuances, and variations in how diagnoses are documented across hospitals could introduce bias. If misclassification of CVD or HDP occurred nondifferentially, this would likely bias our estimates toward the null, potentially underestimating the strength of associations. Conversely, differential misclassification—for instance, if more severe cases are more reliably coded—could lead to overestimation of effects. Thus, our findings should be interpreted as conditional on the accuracy of diagnosis coding.

Administrative database analyses are limited in that chart reviews cannot be performed to adjudicate diagnoses or examine other factors that may have influenced gestational age at delivery such as timing of HDP diagnosis, severity of CVD, worsening of medical comorbidities, cardiac decompensation, or fetal status. Although we suspect patients with underlying CVD who develop HDP are deferred expectant management and thus deliver earlier, there may be differences in the timing of onset of HDP in those with CVD, that we are unable to ascertain. Furthermore, we are unable to examine specific diagnostic factors from catheterizations or echocardiography that would indicate severity of cardiac disease. Future studies with access to disease severity metrics are needed to examine the contribution of disease severity to pre-eclampsia and prematurity risk. Lastly, we were unable to include prior pre-eclampsia as a covariate in our analysis, a key risk factor for pre-eclampsia.^1^^,^^14^ Future studies should aim to quantify the incremental risk that prior or multiple adverse pregnancy outcomes confer on the likelihood of subsequent complications in patients with CVD.

## Conclusions

Our study highlights that the odds of HDP are not uniform across CVD subtypes. We found that HDP significantly affected gestational age at delivery in patients with CVD. Patient counseling should be tailored by CVD subtype to ensure that individuals are informed about the odds of HDP and delivering at an earlier gestational age specific to their CVD subtype.

Future studies should similarly attempt to disaggregate data by CVD subtype, incorporate the timing of HDP diagnosis, and assess morbidity through delivery and the first year postpartum to better inform patient counseling and tailored care.

Perspectives **COMPETENCY IN MEDICAL KOWLEDGE:** In a cohort of pregnant patients with CVD, the risk of HDP varies by subtype, with cardiomyopathy and pulmonary HTN conferring the highest risk of HDP and early delivery.

**TRANSLATIONAL OUTLOOK:** Our large observational study of obstetric patients with CVD supports expanding current guidelines to include cardiomyopathy and pulmonary HTN as high-risk indications for low-dose aspirin prophylaxis.

## Funding support and author disclosures

Dr Federspiel was supported by the 10.13039/100009633Eunice Kennedy Shriver National Institute of Child Health and Human Development of the National Institutes of Health under award number K12HD103083. All other authors have reported that they have no relationships relevant to the contents of this paper to disclose.
