# Supplementary material for: Hypertensive Disorders of Pregnancy in Patients With Cardiac Disease: Risk and Impact on Delivery Timing
Source: JACC Adv. 2025 Sep 15;4(10):102130. doi: 10.1016/j.jacadv.2025.102130 (PMC12466231; doi:10.1016/j.jacadv.2025.102130)
Supplement: Supplemental_Material [file mmc1.docx]

**Supplemental Table 1: ICD Code for Cardiac Disease by subtype**

| Cardiac disease (Leonard codes: I05-I09, I11-I13, I15, I16, I20, I25, I27.8, I30- I41, I44-I49, I50.22, I50.23, I50.32, I50.33, I50.42, I50.43, I50.812, I50.813 O99.41, O99.42, Q20-Q24 (broader set of codes)) | | | Divided into etiology groups below |
| --- | --- | --- | --- |
|  | Secondary hypertension | I15 |  |
|  | Hypertensive crisis | I16 |  |
|  | Pulmonary hypertension | I27.0, I27.2  I27.0, I27.20- 24, I27.29, I27.81-82, I27.89, and I27.9 |  |
|  | Eisenmenger syndrome | I27.83 |  |
|  | Congenital heart disease (CDH) | Q20.*, Q21.* Q21.1, Q21.0, Q21.2, Q21.3, Q24.3, Q25.0, Q26.2, Q26.3, Q26.4, Q21.4, Q21.8, Q21.9, Q22.5, Q22.6, Q24, Q24.5, Q24.8, Q24.9, Q25.48, Q25.5, Q25.6, Q25.7, Q25.71, Q25.72, Q25.79, Q25.8, Q25.9, Z87.79, Q87.89, Q23.4, Q20.8, Z87.74, Q21.3, Q26.2, Q20.0, Q20.1, Q20.2, Q20.3, Q20.4, Q20.5, Q20.9 |  |
|  | Valvular heart disease (VD) | Q23.*, Q23.1, I37.0, Q22.1, Q22.3, I34.1, I05-I09, I36, I34.0, I34.8, I34.9, I35.1, I35.8, I35.9, Q22.0, Q22.2, Q22.3, Q22.8, Q22.9, Q23.0, Q23.1, Q23.2, Q23.3, Q23.8, Q23.9, I05.1, I05.8, I05.9, I06.1, I06.8, I06.9, I05.0, I05.2, I34.2, I34.2, I06.0, I06.2, I35, I35.0, I35.2, Q24.4, Q22.4, Z95.2, Z95.4, Z95.3, Q22.9, |  |
|  | Cardiomyopathy (CM) | I42.0, I42.1, I42.2, I42.3, I42.4, I42.5, I42.6, I42.7, I42.8, I42.9, I51.81, I51.7, O90.3, I50.1, I50.2, I50.20, I50.21, I50.22, I50.23, I50.4, I50.40, I50.41, I50.42, I50.43, I50.8, I50.81, I50.810, I50.811, I50.812, I50.813, I50.814, I50.82, I50.83, I50.84, I50.89, I50.9 |  |
|  | HFpEF (can be included in CM) | I50.3, I50.30, I50.31, I50.32, I50.33 |  |
|  | MI/CAD (ischemic) | I51.1, I51.2, I51.3, I51.5, I20, I24, I25.10, I25.1, I25.11, I25.110, I25.111, I25.118, I25.119, I25.2, I25.42, I25.84, I25.700, I25.701, I25.708, I25.709, I25.710, I25.711, I25.718, I25.719, I25.720, I25.721, I25.728, I25.729, I25.758, I25.790, I25.791, I25.798, I25.799, I25.810, I51.0, I23.0, I23.1, I23.2, I23.3, I23.4, I23.5, I23.6, I23.7, I23.8 |  |
|  | Pericarditis/myocarditis | I30.*, I31.*, I32, I33.*, I40.*, I41.*, I51.4 |  |
|  | Endocarditis | I38, I39.* |  |
|  | Aortopathy | Q25.1, Q87.4, Q87.40, Q87.41, Q87.418, Q87.42, Q87.43, Q25.2, Q25.21, Q25.29, Q25.3, Q25.4, Q25.40, Q25.41, Q25.42, Q25.43, Q25.44, Q25.45, Q25.46, Q25.47, Q25.49, I71.2, I71.4, I71.6, I71.9, I77.81, I77.810, I77.811, I77.812, I77.819, Q87.41, Q87.410, Q87.89, Q79.60, Q79.61, Q79.63 |  |
|  | Afib/aflutter | I48, I48.0, I48.1, I48.2, I48.20, I48.21, I48.3, I48.4, I48.9, I48.91, I48.92 |  |
|  | Arrythmia not afib/flutter | I44.*, I49.1, I49.40, I49.49, I49.3, I49.2, I49.40, I49.49, I49.5, I49.8, I49.9, I47.2, I45.81, Q24.6, Z95.0, Z95.810, I47.0, I47.1, I47.9, I45.6 |  |
|  | Hypertensive heart disease | I51.89 |  |
|  | Other cardiac diseases | I27.1, I51.8, I51.9, Z94.1 |  |

**Supplemental Table 2: ICD10 Codes for Covariates for Outcomes**

|  | **Leonard, et al, 2020**  ICD-10-CM Codes | Adjustment |
| --- | --- | --- |
| **Consistent with Leonard et al** |  |  |
| Gestational diabetes mellitus | O24.4 |  |
| HIV/AIDS | O98.7, B20 |  |
| Previous cesarean birth | O34.21 |  |
| Twin/multiple pregnancy | O30, O31, Z37.2-Z37.7 |  |
| Asthma, acute or moderate/severe | O99.5, J45.21, J45.22,  J45.31, J45.32, J45.4, J45.5, J45.901, J45.902 |  |
| Bleeding disorder, preexisting | D66-D69 |  |
| BMI at delivery | Z68.4 (categorized as ≥40) |  |
|  |  |  |
| Chronic hypertension | O10, O11, I10 |  |
| Chronic renal disease | O26.83, I12, I13, N03-  N05, N07, N08, N11.1, N11.8, N11.9, N18, N25.0, N25.1, N25.81, N25.89, N25.9, N26.9 |  |
| Connective tissue or autoimmune disease | M30-M36 |  |
| Placenta previa, complete or partial | O44.03, O44.13, O44.23, O44.33, |  |
| Substance use disorder | F10-F19, O99.31, O99.32 |  |
| Maternal age |  |  |
| Anemia, preexisting | O99.01, O99.02, D50, D55, D56, D57.1,  D57.20, D57.3, D57.40, D57.80, D55, D56, D58, D59 (limited to codes that are likely present on delivery admission) |  |
| Bariatric surgery | O99.84 |  |
| Gastrointestinal disease | K (entire block), O99.6,  O26.6 |  |
| Major mental health disorder | O99.34, F20-F39 |  |
| Neuromuscular disease | O99.35, G40, G70 |  |
| Placental abruption | O45 |  |
| Placenta accreta spectrum | O43.2 |  |
| Thyrotoxicosis | E05 |  |
|  |  |  |
| **Modified** |  |  |
| Secondary hypertension | I15 |  |
| Hypertensive crisis | I16 |  |
| Preterm birth | Z3A.20-Z3A.36 (<37 weeks) |  |
| Preeclampsia with severe features | O14.1, O14.2, O11 | Excluded as covariates |
| Preeclampsia without severe features or gestational | O13, O14.0, O14.9 | Excluded as covariates |

**Supplemental Table 3: Coding criteria to identify delivery hospitalizations and mode of delivery**

| **Delivery type** | | | **Codes** |
| --- | --- | --- | --- |
| Vaginal delivery | | | O80, 10E0XZZ, 0W8NXZZ |
| Operative vaginal | Forceps deliveries | | 10D07Z3, 10D07Z4, 10D07Z5 |
|  | Vacuum extraction | | 10D07Z6 |
| Cesarean delivery | | | 10D00Z0, 10D00Z1, 10D00Z2 |
|  | | | |
| Cesarean delivery indication codes | | Indicates Intrapartum cesarean delivery |  |
| Previous cesarean delivery | | No | O34.21* |
| Fetal malpresentation | | No | O32.* |
| Fetal hydrocephalus or central nervous system malformation | | No | O33.6*, O35.0* |
| Fetal distress | | Yes | O68, O76, O77.8, O77.9 |
| Failed operative delivery | | Yes | O66.5 |
| Cord Prolapse | | Yes | O69.0* |
| Vasa previa | | No | O69.4* |
| Fetal-Maternal disproportion | | Yes | O33.1*-O33.5*, O33.7*-O33.9* |

**Supplemental Table 4. Subgroup Analysis with cohort stratified by chronic hypertension status: Gestational age at delivery by hypertensive disorder of pregnancy in those with cardiac disease, by subtype**

|  | **With chronic HTN** | | | **Without chronic HTN** | | | |
| --- | --- | --- | --- | --- | --- | --- | --- |
| **Cardiac Disease subtype** | **Adjusted mean gestational age (95% CI), weeks** | **Difference**  **(No HDP – HDP)** | **p-value** | **Adjusted mean gestational age (95% CI), weeks** | **Difference**  **(No HDP – HDP)** | **p-value** |  |
| No cardiac disease |  |  |  |  |  |  |  |
| No HDP | 37.50 (37.47, 37.54) | (reference) |  | 38.50 (38.48, 38.52) | (reference) |  |  |
| Gestational HTN | N/A | N/A | N/A | 38.05 (38.03, 38.07) | 0.45 (0.44, 0.46) | <0.001 |  |
| Preeclampsia | 35.51 (35.47, 35.55) | 1.99 (1.96, 2.03) | <0.001 | 37.55 (37.53, 37.57) | 0.96 (0.95, 0.97) | <0.001 |  |
| Preeclampsia with SF | 34.05 (33.98, 34.13) | 3.45 (3.38, 3.52) | <0.001 | 35.60 (35.58, 35.63) | 2.90 (2.89, 2.91) | <0.001 |  |
| Aortic pathologies |  |  |  |  |  |  |  |
| No HDP | 37.28 (36.72, 37.83) | (reference) |  | 37.86 (37.74, 37.99) | (reference) |  |  |
| Gestational HTN | N/A | N/A | N/A | 37.33 (36.95, 37.72) | 0.53 (0.13, 0.93) | 0.010 |  |
| Preeclampsia | 35.79 (34.84, 36.74) | 1.49 (0.39, 2.58) | 0.013 | 37.20 (36.55, 37.85) | 0.66 (0.00, 1.32) | 0.051 |  |
| Preeclampsia with SF | 33.75 (31.27, 36.22) | 3.53 (1.00, 6.07) | 0.011 | 33.78 (33.20, 34.36) | 4.08 (3.49, 4.68) | <0.001 |  |
| Ischemia |  |  |  |  |  |  |  |
| No HDP | 36.47 (36.16, 36.78) | (reference) |  | 37.59 (37.48, 37.70) | (reference) |  |  |
| Gestational HTN | N/A | N/A | N/A | 37.75 (37.40, 38.10) | -0.15 (-0.52, 0.21) | 0.405 |  |
| Preeclampsia | 33.86 (33.42, 34.31) | 2.61 (2.07, 3.15) | <0.001 | 36.77 (36.26, 37.27) | 0.83 (0.32, 1.34) | 0.002 |  |
| Preeclampsia with SF | 33.36 (32.48, 34.23) | 3.11 (2.19, 4.04) | <0.001 | 34.08 (33.74, 34.43) | 3.51 (3.15, 3.87) | <0.001 |  |
| Congenital disorders |  |  |  |  |  |  |  |
| No HDP | 36.82 (36.49, 37.15) | (reference) |  | 37.99 (37.93, 38.04) | (reference) |  |  |
| Gestational HTN | N/A | N/A | N/A | 37.61 (37.42, 37.80) | 0.38 (0.18, 0.58) | 0.000 |  |
| Preeclampsia | 34.41 (33.86, 34.95) | 2.41 (1.78, 3.05) | <0.001 | 37.53 (37.25, 37.81) | 0.45 (0.17, 0.74) | 0.002 |  |
| Preeclampsia with SF | 34.35 (32.82, 35.88) | 2.47 (0.91, 4.03) | 0.003 | 34.65 (34.39, 34.91) | 3.34 (3.08, 3.60) | <0.001 |  |
| Pulmonary HTN |  |  |  |  |  |  |  |
| No HDP | 35.76 (35.15, 36.36) | (reference) |  | 36.58 (36.38, 36.77) | (reference) |  |  |
| Gestational HTN | N/A | N/A | N/A | 36.22 (35.63, 36.80) | 0.36 (-0.25, 0.98) | 0.247 |  |
| Preeclampsia | 32.29 (31.69, 32.90) | 3.46 (2.61, 4.32) | <0.001 | 35.19 (34.60, 35.77) | 1.39 (0.78, 2.01) | <0.001 |  |
| Preeclampsia with SF | 32.42 (30.92, 33.93) | 3.33 (1.71, 4.95) | <0.001 | 33.62 (33.26, 33.98) | 2.96 (2.55, 3.37) | <0.001 |  |
| Cardiomyopathy |  |  |  |  |  |  |  |
| No HDP | 35.76 (35.55, 35.97) | (reference) |  | 36.80 (36.72, 36.89) | (reference) |  |  |
| Gestational HTN | N/A | N/A | N/A | 36.83 (36.56, 37.09) | -0.02 (-0.30, 0.25) | 0.864 |  |
| Preeclampsia | 33.18 (32.93, 33.44) | 2.58 (2.25, 2.91) | <0.001 | 35.90 (35.62, 36.18) | 0.90 (0.61, 1.19) | <0.001 |  |
| Preeclampsia with SF | 31.86 (31.37, 32.35) | 3.90 (3.37, 4.43) | <0.001 | 33.37 (33.20, 33.54) | 3.43 (3.24, 3.62) | <0.001 |  |
| Valvular disease |  |  |  |  |  |  |  |
| No HDP | 37.01 (36.78, 37.23) | (reference) |  | 38.11 (38.06, 38.16) | (reference) |  |  |
| Gestational HTN | N/A | N/A | N/A | 37.80 (37.64, 37.95) | 0.31 (0.15, 0.47) | 0.000 |  |
| Preeclampsia | 34.28 (33.97, 34.58) | 2.73 (2.35, 3.10) | <0.001 | 36.86 (36.64, 37.08) | 1.25 (1.03, 1.47) | <0.001 |  |
| Preeclampsia with SF | 32.95 (32.30, 33.60) | 4.05 (3.37, 4.74) | <0.001 | 34.61 (34.44, 34.77) | 3.50 (3.34, 3.67) | <0.001 |  |

**Supplemental Table 5. Subgroup Analysis with cohort stratified by chronic hypertension status : Adjusted Odds Ratio of Hypertensive disorder of pregnancy by cardiac disease subtype**

|  | **With chronic HTN** | | **Without chronic HTN** | | |
| --- | --- | --- | --- | --- | --- |
| **Cardiac Disease subtype** | **Odds of HDP* [OR (95% CI)]** | **p-value** | **Odds of HDP* [OR (95% CI)]** | **p-value** |  |
| Aortic pathology | 0.70 (0.46, 1.06) | 0.087 | 0.90 (0.69, 1.18) | 0.448 |  |
| Ischemia | 0.87 (0.70, 1.09) | 0.222 | 1.09 (0.90, 1.33) | 0.373 |  |
| Congenital | 0.64 (0.49, 0.83) | 0.001 | 0.88 (0.78, 0.99) | 0.032 |  |
| Pulmonary hypertension | 1.21 (0.84, 1.73) | 0.305 | 1.76 (1.38, 2.24) | <0.001 |  |
| Cardiomyopathy | 0.96 (0.83, 1.10) | 0.541 | 2.21 (1.98, 2.46) | <0.001 |  |
| Valvular disease | 1.03 (0.88, 1.21) | 0.716 | 1.18 (1.08, 1.29) | <0.001 |  |

**Supplemental Discussion of Subgroup Analysis:**

In our primary analysis we treated cHTN as a covariate in our primary model to isolate the effect of CVD on the development of hypertensive disorders of pregnancy and to account for cHTN as a potential confounder.

Per reviewer comments, we conducted a subgroup analysis to explore whether the association between CVD subtypes and hypertensive disorders of pregnancy varied by comorbid chronic hypertension (cHTN), we conducted a subgroup analysis stratified by cHTN status. Among individuals without cHTN, several CVD subtypes—including cardiomyopathy, pulmonary hypertension, and valvular disease—were significantly associated with increased odds of hypertensive disorders of pregnancy. In contrast, these associations were attenuated or non-significant in the cHTN subgroup, suggesting that the elevated baseline risk conferred by cHTN may obscure the additional contribution of CVD to the risk of hypertensive disorders of pregnancy. These findings suggest that in the absence of an already elevated baseline risk, the impact of underlying CVD on hypertensive disorders of pregnancy becomes more pronounced. This highlights the importance of considering CVD as an independent risk factor for hypertensive disorders of pregnancy, particularly in patients who may not otherwise be classified as high-risk based on traditional factors such as chronic hypertension.

When examining gestational age at delivery, stratified by cHTN, patients with CVD and preeclampsia with severe features consistently delivered at earlier gestational ages in the cHTN subgroup compared to those without cHTN, reflecting their greater baseline obstetric risk. Similarly, among those with preeclampsia without severe features, individuals with cHTN also delivered earlier than their non-cHTN counterparts. This pattern suggests that cHTN may contribute to earlier onset, more rapid clinical progression, or lower thresholds for obstetric intervention, even in the absence of severe disease features.

However, the magnitude of the difference in gestational age between those with and without hypertensive disorders of pregnancy varied by cardiac disease subtype. Among patients without cHTN, the relative impact of hypertensive disorders of pregnancy on delivery timing was more pronounced for aortic pathologies, ischemic heart disease, and congenital heart disease, likely due to a later baseline gestational age. In contrast, for pulmonary hypertension, cardiomyopathy, and valvular disease, the difference in gestational age associated with hypertensive disorders of pregnancy was similar or greater in the cHTN subgroup, suggesting a compounded effect of multiple comorbidities.
